# Editorial: The use of Structural Equation Modeling (SEM) methods in eating behavior research

**DOI:** 10.3389/fpsyg.2024.1378515

**Published:** 2024-02-19

**Authors:** Edward Wilson Ansah, Daniel Rodriguez, C. Blair Burnette

**Affiliations:** ^1^Department of Health, Physical Education and Recreation, University of Cape Coast, Cape Coast, Ghana; ^2^Department of Urban Public Health and Nutrition, La Salle University, Philadelphia, PA, United States; ^3^Department of Psychology, University of Minnesota, St. Paul, MN, United States

**Keywords:** Structural Equation Modeling, eating disorders, nutrition, statistical analysis, data, questionnaire

Malnutrition, i.e., under-nutrition and over-nutrition, in terms of overweight and obese keep rising in all segments of the population, but disproportionately affecting children and adolescents. The evidence is that many factors including parental feeding practices, personality traits of the person, food availability and preparation among others influence the eating behavior of the person which in turn affects their nutrition status. Moreover, the eating disorder literature lacks standardized instruments to measure the interactions of these complex variables. Therefore, twenty-first century statistics like SEM and its closely related tools become essential to explore and provide understanding of these variables. The aim of this special collection is to collate research evidence that seek to explore eating behavior using a Structural Equation Modeling (SEM) framework. These articles used multivariate methods such as confirmatory factor analysis (CFA), exploratory SEM, latent growth curve modeling (LGCM), and latent variable mixture modeling (LVMM) methods including growth mixture modeling (GMM) and sequential processes GMM. The collection also aimed at topics such as testing the fit of theoretical models of eating behavior to the data, assessment of change in eating behavior across time, confirming the validity of an existing measure, mediation and moderation models, or assessing for heterogeneity in eating behaviors, with either longitudinal or cross-sectional studies.

This study, Pesenti et al. compared the variable selection ability of three Bayesian variable selection models; Bayesian Kernel Machine Regression (BKMR), Bayesian Semiparametric Regression (BSR), and Bayesian Least Absolute Shrinkage and Selection Operator (BLASSO) by applying simulated and real data. Using the R programming language analyses, the models' goodness of fit was evaluated by simulating several predictors with a complex correlation structures under different sample sizes. Furthermore, the models' performance was assessed on a primary data from weight loss in hospitalized obese women from the Follow Up obese patients at AUXOlogico Institute (FUOBAUXO) cohort. The aim was also to determine the association between biochemical, anthropometric, and clinical variables on weight loss percentage in these patients over a period of 40 days. These Bayesian approaches need to be applied under different scenarios, dependent on the sample sizes of the dataset, the correlation structure, and the predictor-outcome relationships. Thus, for large sample size data, BKMR and BSR may be employed randomly when the dataset is large enough to capture complex relationships between predictors and the outcomes even when the variables are highly correlated. BKMR could correctly select the important variables even with small sample size data. Moreover, BLASSO may be a better approach when it is reasonable to hypothesize the absence of interactions between predictors. In the case of obese women, all the models (BKMR, BSR, and BLASSO) identified the lean body mass of the women, a situation which underscores the importance of body composition in the process of weight loss.

## Author contributions

EA: Writing – original draft, Writing – review & editing. DR: Writing – review & editing. CB: Writing – review & editing.

